# Characterization of the chloroplast genome of the marine microalga *Tetraselmis marina* (Cienkowski) R.E.Norris, Hori & Chihara 1980

**DOI:** 10.1080/23802359.2023.2288892

**Published:** 2023-12-11

**Authors:** Fangfang Yang, Yi Huang, Lijuan Long

**Affiliations:** a Key Laboratory of Tropical Marine Bio-resources and Ecology, South China Sea Institute of Oceanology, Chinese Academy of Sciences, Guangzhou, China; b Southern Marine Science and Engineering Guangdong Laboratory, Guangzhou, China

**Keywords:** *Chloroplast genome*, marine microalga, *Tetraselmis marina*

## Abstract

*Tetraselmis marina* (Cienkowski) R.E.Norris, Hori & Chihara 1980, a costal green microalga, is considered as a promising animal feed in aquaculture due to the high content of fatty acids and carotenoid. Furthermore, *T. marina* plays important roles in bioremediation. In this study, we assembled the complete chloroplast genome of *T. marina*. Results showed that the full length of the complete chloroplast genome was 96,151 bp, containing a large single-copy region of 62,574 bp, a small single-copy region of 1261 bp, and a pair of inverted repeat regions of 16,158 bp. The GC content of the genome was 36.6%. A total of 125 genes were annotated, including 81 protein coding genes, 38 tRNA genes, and six rRNA genes. Phylogenetic analysis based on 22 chloroplast genomes suggested that *T. marina* was closely related to *Tetraselmis* sp. CCMP 881.

## 1. Introduction

*Tetraselmis marina* (Cienkowski) R.E.Norris, Hori & Chihara 1980, a common coastal microalga, belongs to genus Tetraselmis (Chlorodendraceae). *Tetraselmis* sp. is commonly considered as a promising potential source of antioxidants or animal feed due to the high contents of fatty acid and carotenoid profile (Moussa et al. 2017; Oliveira Moser et al. 2022). Additionally, several species of *Tetraselmis* sp. are commonly potential candidates for radioactive Sr bioremediation (Fukuda et al. 2014). Interestingly, several species of *Tetraselmis* sp. possess biomineralization capacity, and can produce intracellular inclusions of amorphous calcium carbonate (i.e. micropearls, Martignier et al. 2018, 2020). It implies that *Tetraselmis* sp. may play a role in the ocean carbon cycle. However, the information about the chloroplast genome of *T. marina* has been not reported. In the present study, we sequenced and assembled the chloroplast genome of *T. marina*, and analyzed its phylogenetic position.

## 2. Materials

**Figure 1.**
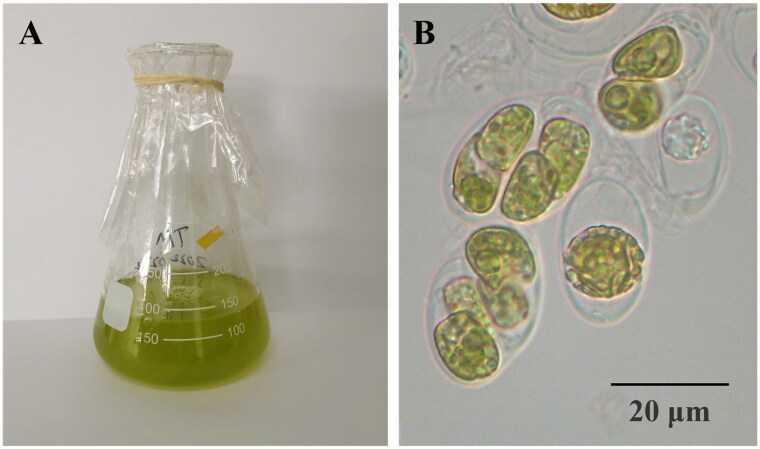
The morphology of *T. marina* (A) in flasks and (B) under a microscope. Photograph was taken by Fangfang Yang. It is a unicellular with a size range between 10 and 20 μm.

*T. marina* was isolated from coral *Pocillopora damicornis* in the coast of Sanya City, China (18°18′ N and 109°48′ E), and cultured in f/2 medium (Figure 1). It was deposited at the laboratory of South China Sea Institute of Oceanology, Chinese Academy of Sciences, Guangzhou City, Guangdong Province (http://www.scsio.ac.cn/, Fangfang Yang, ycuyang@scsio.ac.cn) under voucher number SY20210603.

## 3. Methods

Whole-genome DNA was extracted according to a modified CTAB protocol (Doyle 1987). The lysis incubation was changed from 30 min to 60 min and then 2 μL RNase A was added at 37 °C for 30 min. The purified genomic DNA was sheared into 350 bp fragments to construct a paired-end (PE) library according to the Nextera XT sample preparation procedures (Illumina, San Diego, CA). The PE reads of 150 bp were generated using a Novaseq 6000 sequencer (Illumina, San Diego, CA) (Fig. S1). A total of 4.15 G of raw data was obtained for further analysis. The GC content, Q20 value and Q30 value of the clean data were 54.94%, 96.53%, and 91.09%, respectively. High-quality reads were assembled into the chloroplast genome using the *de novo* assembler SPAdes v.3.14.1 software. Finally, the PGA program was used to annotate the chloroplast genome (Qu et al. 2019), using the chloroplast genome of *Tetraselmis* sp. CCMP 881 (GenBank accession number KU167097.1) as the reference. In order to identify the phylogenetic relationship of *T. marina*, the 12 common protein-coding genes in each complete mitochondrial genome of 22 chloroplast genomes of related marine microalga species were aligned using the MAFFT version 7 software with the FFT-NS-2 strategy (Katoh and Standley 2016). Then, a phylogenetic tree was conducted based on the maximum-likelihood method using 1000 bootstrap replicates by IQ-TREE 2.0 (Nguyen et al. 2015). *Oltmannsiellopsis viridis* (GenBank accession number DQ291132.1) was used as an outgroup species.

## 4. Results

**Figure 2.**
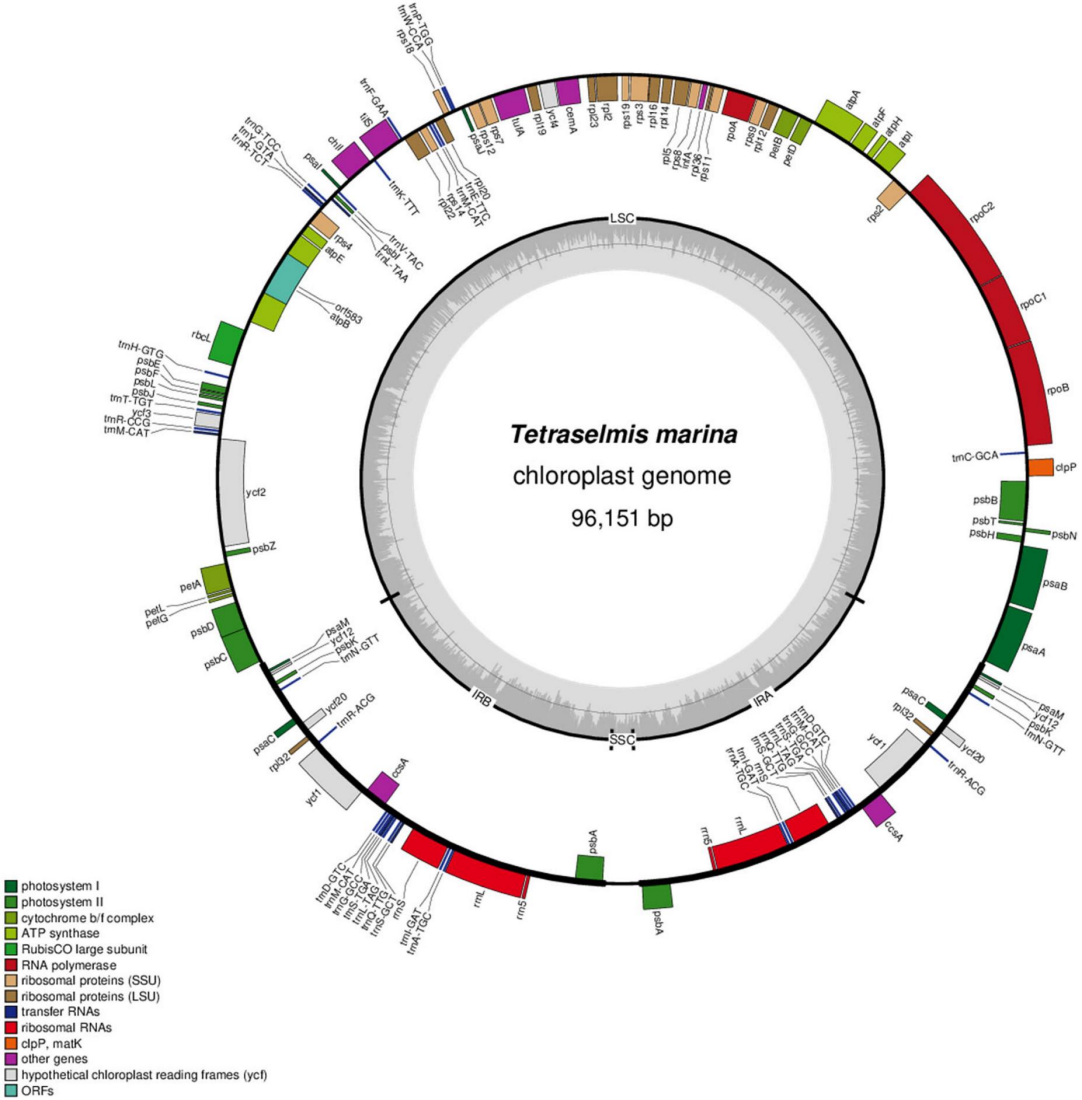
Gene map of the complete chloroplast genome of *T. marina* using OGDRAW (https://chlorobox.mpimp-golm.mpg.de/OGDraw.html). Genes shown on the outside of the circle are transcribed clockwise, while those inside are transcribed counterclockwise. Arrangement of 125 genes represented in the map, including 81 protein-coding genes, six rRNA, and 38 tRNA genes.

**Figure 3.**
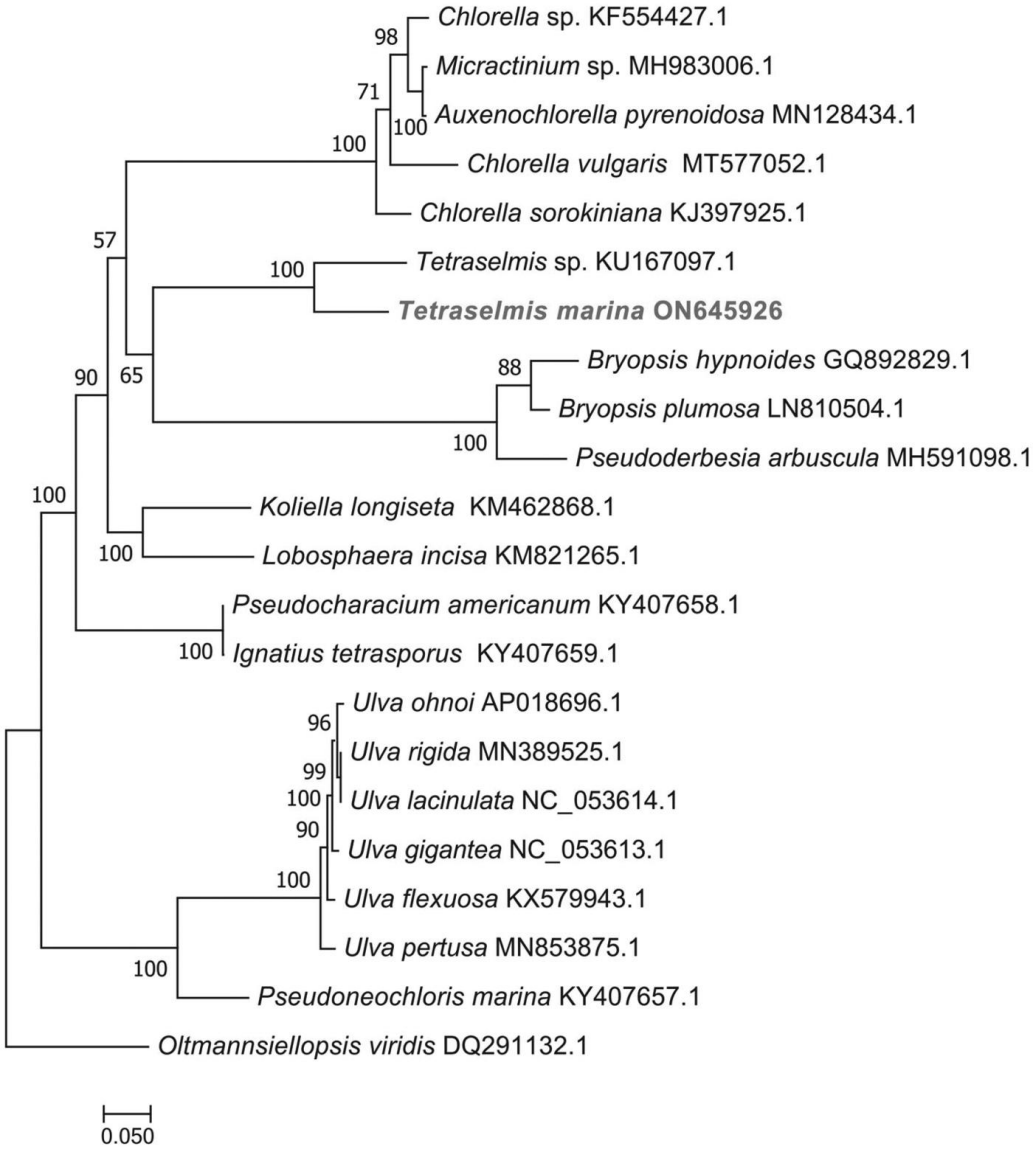
Maximum-likelihood (ML) phylogenetic tree based on complete chloroplast genomes. *Oltmannsiellopsis viridis* was used as an outgroup species. The numbers on each branches indicate the boot support value of the ML analyses. The scale bar was 0.050. The following sequences were used: *Chlorella* sp. KF554427.1, *Micractinium* sp. MH983006.1, *Auxenochlorella pyrenoidosa* MN128434.1, *Chlorella vulgaris* MT577052.1, *Chlorella sorokiniana* KJ397925.1, *Tetraselmis* sp. CCMP 881 KU167097.1 (Turmel et al. 2020), *Bryopsis hypnoides* GQ892829.1, *Bryopsis plumosa* GenBank: LN810504.1, *Pseudoderbesia arbuscula* MH591098.1, *Koliella longiseta* KM462868.1, *Lobosphaera incisa* KM821265.1, *Pseudocharacium americanum* KY407658.1, *Ignatius tetrasporus* KY407659.1, *Ulva ohnoi* AP018696.1, *Ulva rigida* MN389525.1, *Ulva lacinulata* NC_053614.1, *Ulva gigantea* NC_053613.1, *Ulva flexuosa* KX579943.1, *Ulva pertusa* MN853875.1, *Pseudoneochloris marina* KY407657.1, and *Oltmannsiellopsis viridis* DQ291132.1 (Pombert et al. 2006).

The complete chloroplast genome sequence of *T. marina* was submitted to GenBank under accession number ON645926. The length of chloroplast genome sequence of *T. marina* was 96,151 bp, consisting of two inverted repeat regions of 16,158 bp, separated by a large single-copy region of 62,574 bp, and a small single-copy region of 1261 bp (Figure 2). The overall GC content was 36.6%. There is a cis-splicing gene called atpB (Figure S2). A total of 125 genes were annotated, consisting of 81 protein-coding genes, six rRNA, and 38 tRNA genes. Phylogenetic analysis results showed that *T. marina* was closely related to *Tetraselmis* sp. CCMP 881 (Figure 3).

## 5. Discussion and conclusions

In this study, the complete chloroplast genome of *T. marina* was assembled and annotated for the first time. It was 96,151 bp, containing a large single-copy region of 62,574 bp, a small single-copy region of 1,261 bp, and a pair of inverted repeat regions of 16,158 bp. Compared with the chloroplast genome of the marine microalga previously published data, this result indicated that the chloroplast genome of *T. marina* showed a high level of gene synteny with one publicly available *Tetraselmis* sp. CCMP 881 (Turmel et al. 2020). Phylogenetic trees analysis provided new insight into the genetic relationship of *T. marina*. Further investigations are necessary to understand and document the evolution of the genus *Tetraselmis*.

## Supplementary Material

Supplemental Material

## Data Availability

The genome sequence data that support the findings of this study are openly available in GenBank of NCBI at https://www.ncbi.nlm.nih.gov/ under the accession no. ON645926. The associated BioProject, SRA, and Bio-Sample numbers are PRJNA844566, SRR19521163, and SAMN28834016, respectively.
