# Supplementary material for: Characterization of the chloroplast genome of the marine microalga Tetraselmis marina (Cienkowski) R.E.Norris, Hori & Chihara 1980
Source: Mitochondrial DNA B Resour. 2023 Dec 11;8(12):1347–50. doi: 10.1080/23802359.2023.2288892 (PMC10776064; doi:10.1080/23802359.2023.2288892)
Supplement: Supplemental Material [file TMDN_A_2288892_SM6650.doc]

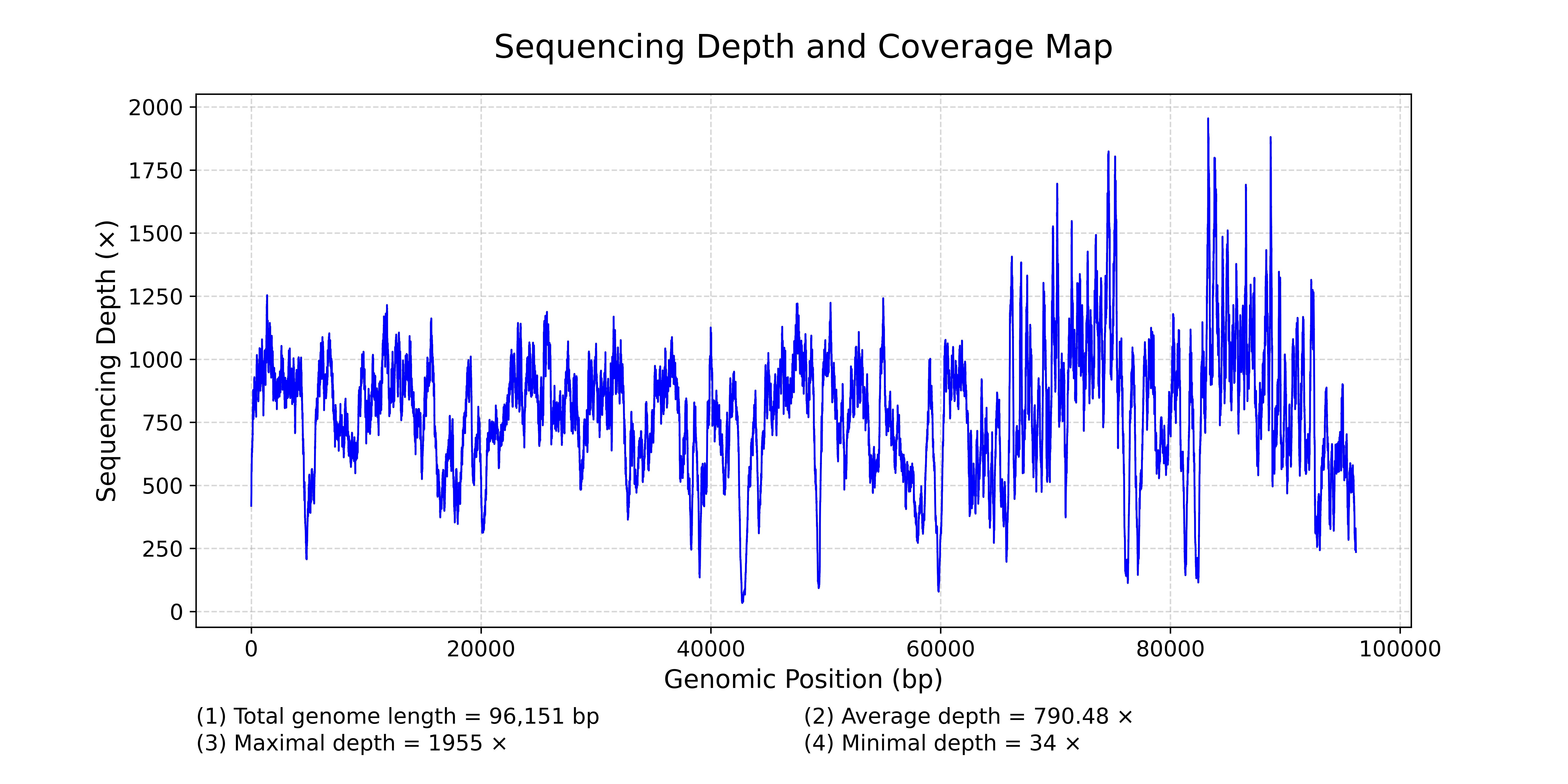


Fig S1 Coverage depth figure of *T. marina*.


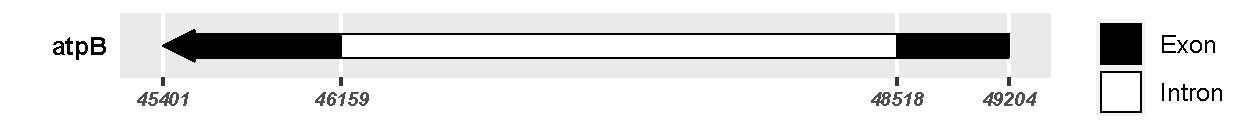


Fig S2 Schematic map of the cis-splicing genes in the *T. Marina* chloroplast genome.
